# MiR-200c overexpression is associated with better efficacy of EGFR-TKIs in non-small cell lung cancer patients with EGFR wild-type

**DOI:** 10.18632/oncotarget.2302

**Published:** 2014-08-08

**Authors:** Jiayu Li, Xuefei Li, Shengxiang Ren, Xiaoxia Chen, Yishi Zhang, Fei Zhou, Mingchuan Zhao, Chao Zhao, Xiu Chen, Ningning Cheng, Yinmin Zhao, Caicun Zhou, Fred R. Hirsch

**Affiliations:** ^1^ Department of Medical Oncology, Shanghai Pulmonary Hospital, Tongji University School of Medicine, Tongji University Medical School Cancer Institute, Shanghai, China; ^2^ Department of Lung Cancer and Immunology, Shanghai Pulmonary Hospital, Tongji University, Tongji University Medical School Cancer Institute, Shanghai, China; ^3^ Department of Respiration, Zaozhuang Municipal Hospital, Zaozhuang, Shandong, China; ^4^ Department of Central Laboratory, Shanghai Pulmonary Hospital, Tongji University, Shanghai, China; ^5^ Departments of Medicine and Pathology, University of Colorado Cancer Center, Aurora, Colorado, USA

**Keywords:** Non-small cell lung cancer, MiR-200c, Epidermal growth factor receptor, Wild type, Tyrosine-kinase inhibitor

## Abstract

Several randomized trials have demonstrated non-small cell lung cancer (NSCLC) patients with activating epidermal growth factor receptor (EGFR) mutations can achieve favorable clinical outcomes on treatment with EGFR tyrosine kinase inhibitors (TKIs). *EGFR* mutation is considered as a predictive marker for efficacy of EGFR-TKIs in NSCLC. Here we show miR-200c overexpression was correlated with the epithelial phenotype and sensitivity to gefitinib in *EGFR* wild-type NSCLC cell lines. Up-regulated miR-200c could regain the sensitivity to gefitinib in the *EGFR* wild-type cell lines and miR-200c could regulate epithelial to mesenchymal transition through PI3K/AKT and MEK/ERK pathways. NSCLC patients at advanced stage (N=150) who received EGFR-TKIs (gefitinib or erlotinib) as second- or third-line therapy from September 2008 to December 2012 were included in the study. In 66 NSCLC patients with wild-type *EGFR*, high levels of miR-200c expression was associated with higher disease control rate (DCR), longer progression-free survival (PFS) and longer overall survival (OS) compared with low miR-200c expression subgroup. In the subgroup with *EGFR* mutation, the trend remained the same but not statistically significant. Overall, these findings indicated that miR-200c might be a predictive biomarker for sensitivity to EGFR-TKIs in advanced NSCLC patients with wild-type *EGFR*.

## INTRODUCTION

Lung cancer is the leading cause of cancer-related mortality worldwide and non-small cell lung cancer (NSCLC) accounts for 80% of lung cancer [1]. Recently, with the identification of several oncogenic drivers, personalized therapy has gained prominence in patients with advanced NSCLC. Patients harboring epidermal growth factor receptor (EGFR) mutation or *ALK/ROS1* fusion may experience unprecedented success on treatment with EGFR tyrosine kinase inhibitors (EGFR-TKIs) or anaplastic lymphoma kinase (ALK) inhibitor [2-4]. Unfortunately, only minority patients have these driver mutations. About 3-5% of NSCLC patients harbor ALK-rearrangement [5-7], and about 30-40% East Asian patients harbor *EGFR* mutation (*EGFR-*MUT) [8-10]. Though the majority of NSCLC patients were *EGFR* wild type (*EGFR*-WT), there are still 3-15% of them respond to EGFR-TKIs with a disease control rate (DCR) of 40-60% [8, 11-13], which suggests that a subgroup of *EGFR*-WT patients benefit from EGFR inhibitor treatment. Therefore, to achieve optimal outcomes, *EGFR*-WT patients who may benefit from EGFR-TKIs treatment should be identified.

Epithelial-to-mesenchymal transition (EMT) is featured as loss of E-cadherin expression and simultaneous increase of mesenchymal biomarkers expression. This in turn enhances tumor cell motility and plays a crucial role in tumor metastasis and drug resistance. Studies have suggested that acquisition of mesenchymal phenotype is associated with acquired chemo-resistance and confer primary resistance to trastuzumab, a monoclonal antibody against HER2/neu receptor [14, 15]. In NSCLC patients, EMT was reportedly associated with resistance to EGFR-TKIs. Further, mesenchymal markers were found to be more frequently expressed in clinical samples of acquired resistance of EGFR-TKIs [16, 17].

MicroRNAs (miRNAs) are small, non-coding RNAs. By binding with 3’-UTR of targeted mRNA, they inhibit or down-regulate protein expression and regulate complex biologic processes, such as cell differentiation, proliferation and apoptosis. miRNAs can act as either oncogenes or tumor suppressor genes in different types of cancers [18, 19]. Recently, miR-200c had been reported to be involved in the resistance of antiestrogen drugs in breast cancer cells [20]. Moreover, it is also found that miR-200c acted as the main suppressor of EMT and was down-regulated in several kinds of tumors including lung cancer [21]. Thus, we hypothesized that miR-200c is associated with the resistance of EGFR-TKIs in NSCLC patients.

To better clarify the function of miR-200c in NSCLC and its association with the efficacy of EGFR-TKIs, we performed a comprehensive analysis of miR-200c expression with EMT status, and its effect on resistance of EGFR-TKIs in NSCLC cell lines. Further, we investigated the feasibility of miR-200c expression to predict the outcomes of EGFR-TKIs in 150 advanced NSCLC patients.

## RESULTS

### MiR-200c was down-expressed in the NSCLC cell lines primarily resistant to EGFR-TKIs

**Figure 1:**
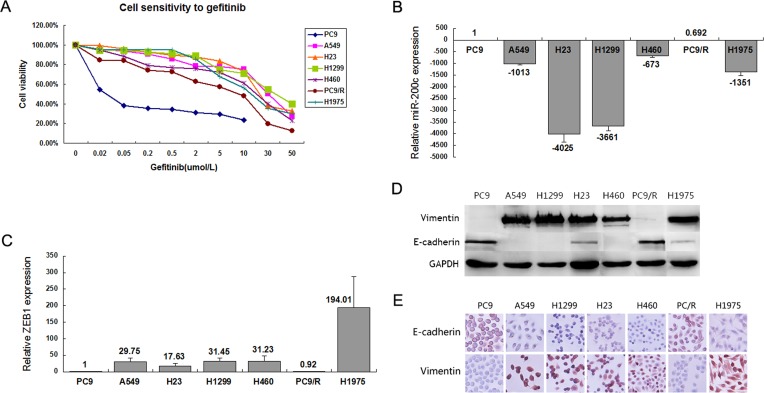
Phenotype and sensitivity to gefitinib in 7 NSCLC cell lines **(A)** Cell proliferation measurement of the 7 NSCLC cell lines when treated with increasing dose of gefitinib. **(B)** Expression of miR-200c was evaluated by qRT-PCR in 7 NSCLC cell lines. **(C)** Expression of ZEB1 was detected by qRT-PCR in 7 NSCLC cell lines. **(D)** Western blot analysis of epithelial marker E-cadherin and mesenchymal marker vimentin in the panel of NSCLC cell lines. **(E)** Immunocytochemistry of epithelial marker E-cadherin and mesenchymal marker vimentin in the panel of NSCLC cell lines. All data are representative of 3 independent experiments.

Seven NSCLC cell lines (PC9, A549, H1299, H23, H460, H1975 and PC9/R) were selected to evaluate their miR-200c expression by qRT-PCR. These cell lines displayed diverse sensitivities to gefitinib according to cell growth inhibition assay by MTT (Figure 1A). Among them, PC9 was a gefitinib-sensitive cell line and PC9/R was an acquired resistant cell line to gefitinib, other 5 cell lines were primarily resistant to gefitinib. The characteristics of cell lines are listed in Supplementary Table S1. The results showed that miR-200c expression was decreased, and ZEB1, the potent target of miR-200c was increased in all 5 primary resistant NSCLC cell lines, compared with PC9 (Figure 1B, 1C).

The expression of E-cadherin and vimentin in 7 NSCLC cell lines were screened using Western blot analysis and immunocytochemistry. The results showed that vimentin was overexpressed, while E-cadherin was downexpressed in primary resistant NSCLC cell lines (Figure 1D and 1E).

### MiR-200c regulated EMT by targeting ZEB1 in NSCLC cells

**Figure 2:**
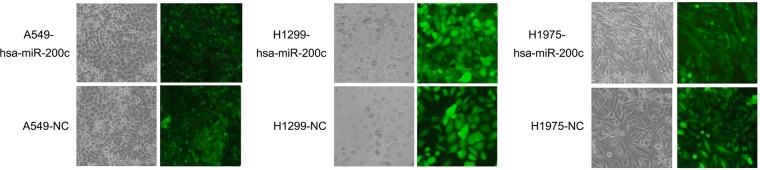
GFP-labeled lentivirus (LV)-hsa-miR-200c and its negative control (NC) infected A549, H1975 and H1299 The infection efficiency is still keeping in the range of 80%-90%.

Expression of miR-200c in A549, H1299 and H1975 cell lines was upregulated through infection with GFP labeled LV-hsa-miR-200c. To restrain miR-200c expression, PC9 cell line was transfected with miR-200c inhibitor. The infection efficiency was about 80%-90% (Figure 2).

**Figure 3:**
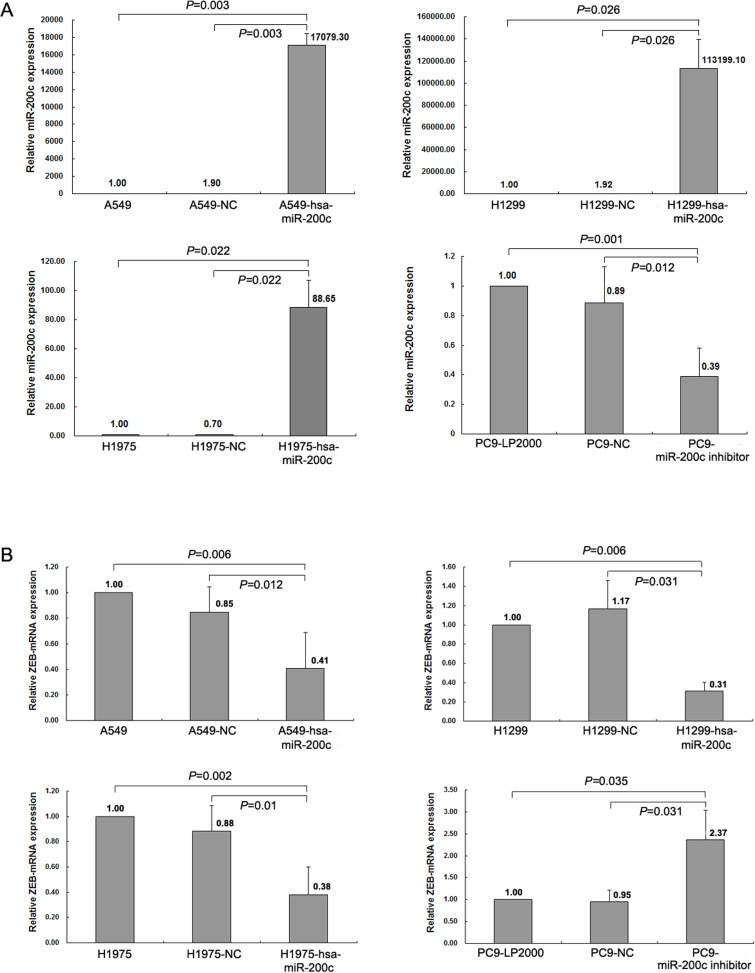
miR-200c regulates ZEB1 expression at the post-transcriptional level **(A)** Expression of miR-200c was determined by qRT-PCR in A549 and H1975 after infected by LV-hsa-miR-200c or NC, and miR-200c expression in PC9 after transfected with miR-200c inhibitor and NC. **(B)** Detection of mRNA expression of ZEB1 in A549, H1975 and PC9 cells after miR-200c was regulated. All data are representative of 3 independent experiments.

**Figure 4:**
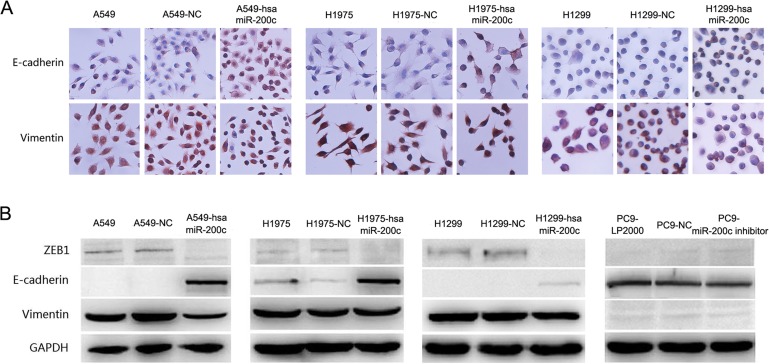
Over expression of miR-200c can restore epithelial phenotype in NSCLC **(A)** Immunocytochemistry staining for E-cadherin and vimentin in A549, H1975 and H1299 after infected by LV-hsa-miR-200c or NC. **(B)** Western blot analysis showed protein expression of EMT markers and ZEB1 in A549, H1975, H1299 and PC9 cells after miR-200c was regulated by LV-hsa-miR-200c or miR-200c inhibitor. All data are representative of 3 independent experiments.

Several studies have proved that mRNA of ZEB1 was the target of miR-200c in different cancers, such as ovarian cancer and bladder cancer [21, 22]. As shown in Figure 3(A, B), LV-hsa-miR-200c upregulated miR-200c expression and mRNA of ZEB1 was reduced in the 3 cell lines. Conversely, treatment of PC9 cell line with miR-200c inhibitor suppressed miR-200c expression and induced up-regulation of mRNA of ZEB1 according to qRT-PCR analysis. Western blot and immunocytochemistry suggested that overexpression of miR-200c led to a significant increase of E-cadherin and apparently decrease of ZEB1 in A549, H1299 and H1975 in protein level, meanwhile, a slight decrease of vimentin was observed (Figure 4A, B). In contrast, NC cells did not display any alteration of vimentin and ZEB1 expressions (Figure 4A, B). A slight decrease of E-cadherin was detected after downregulation of miR-200c by transfection of its inhibitor in PC9 (Figure 4B). However, miR-200c suppression did not change the protein expression of ZEB1 obviously (Figure 4B). This phenomenon can be attributed to very low expression of ZEB1 in PC9, and Western blotting was not sensitive enough to detect protein expression. All the results above suggested that miR-200c was a crucial regulator of EMT by targeting ZEB1, and high-expression of miR-200c might result in mesenchymal-to-epithelial transition (MET). Therefore, it is reasonable to speculate that miR-200c may decrease ZEB1 to inhibit EMT in NSCLC.

### Ectopic expression of miR-200c resulted in partial restoration of gefitinib sensitivity in NSCLC cells

**Figure 5:**
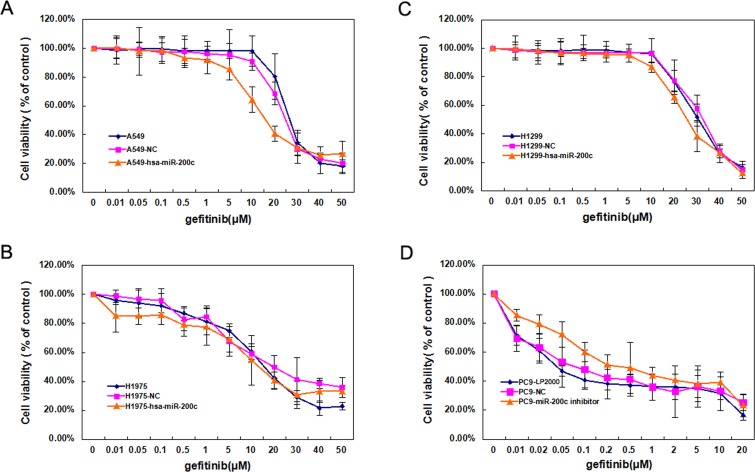
Low expression of miR-200c contributes to gefitinib drug resistance **(A**-**C)** Effects of LV-hsa-miR-200c on gefitinib sensitivity in A549, H1975 and H1299 cells. **(D)** Effects of miR-200c inhibiter on gefitinib sensitivity in PC9 cells. Data are mean ± SD from 3 independent experiments.

We investigated whether miR-200c expression could affect gefitinib sensitivity in the NSCLC cell lines. Interestingly, upregulation of miR-200c increased gefitinib sensitivity in A549 and H1299, but not in H1975 (Figure 5A-5C). *T790M* could be the main cause of acquired resistance to EGFR-TKIs in H1975. MiR-200c could upregulate the expression of E-cadherin and trigger MET in H1975, but cannot reverse the resistance to gefitinib owing to *T790M* existence. Inversely, blocking miR-200c expression of PC9 caused resistance of gefitinib compared with parental and NC cells (Figure 5D).

### EGFR-TKIs resistance induced by miR-200c downexpression was mediated through PI3K/AKT and MEK/ERK pathway

**Figure 6:**
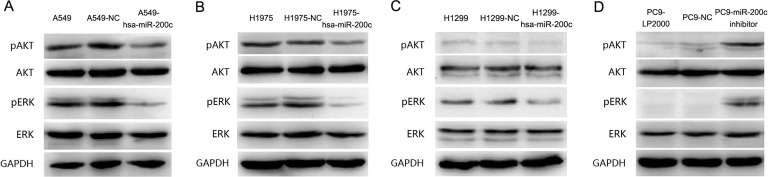
PI3K/AKT and MEK/ERK are two important signal pathways regulated by miR-200c **(A-C)** Western blot analysis of AKT, pAKT, ERK and pERK levels in A549, H1975 and H1299 cells after infected with LV-hsa-miR-200c or NC. **(D)** Western blotanalysis of AKT, pAKT, ERK and pERK levels in PC9 cells after transfected with miR-200c inhibitor or NC. GAPDH is included as a loading control. Results are representative of at least three independent experiments.

PI3K/AKT and MEK/ERK signal pathways are the main downstream pathways of EGFR. To explore the downstream mechanisms of miR-200c mediated in EGFR-TKIs resistance, we detected pAKT and pERK expression before and after LV-hsa-mir-200c infecting A549, H1299 and H1975. We found that pAKT and pERK were repressed when miR-200c was upregulated (Figure 6A-C). Moreover, phosphorylation of AKT and ERK were markedly activated after silencing miR-200c expression in PC9 by miR-200c inhibitor (Figure 6D).

### Patient characteristics

A total of 150 patients with advanced NSCLC were included into this study. The median age was 59 years (range, 30-81 years). The proportions of male patients, ever smokers and patients with adenocarcinoma accounted for 56.0%, 27.3% and 67.3%, respectively. Patients received a median of two prior chemotherapy regimens (range, 1-2 regimens). MiR-200c expression levels were detected in all of the 150 patients. Mutations of *EGFR* were successfully performed in 139 patients, including 73 patients with activated *EGFR* mutation and 66 with *EGFR* wild type, while the other 11 failed the test owing to poor quality of DNA. The expression of miR-200c was significantly lower in *EGFR*-WT groups than *EGFR*-MUT group (0.04802±0.01533 vs. 0.11297±0.04634, P = 0.049). There was no significant association between the miR-200c expression level and other characteristics such as age, gender, smoking status, histological type and so on (Table 1).

**Table 1:** Correlations between baseline characteristics and miR-200c expression in the 150 patients with advanced NSCLC

Characteristic		No. (%) N=150	MiR-200c		p-value
			Low(%) n=83	High(%) n=67	
**Age (years)**	<65	111 (74.0)	64 (42.7)	47 (31.3)	0.334
	≥65	39 (26.0)	19 (12.7)	20 (13.3)	
**Gender**	Female	66 (44.0)	32 (21.3)	34 (22.7)	0.135
	Male	84 (56.0)	51 (34.0)	33 (22.0)	
**Disease stage**	IIIB	20 (13.3)	9 (6.0)	11 (7.3)	0.318
	IV	130 (86.7)	74 (49.3)	56 (37.3)	
**ECOG PS^a^**	0-1	139 (92.7)	75 (50.0)	64 (42.6)	0.347
	2-3	11 (7.3)	8 (5.3)	3 (2.0)	
**Histology**	Adenocarcinoma	101 (67.3)	53 (35.3)	48 (32.0)	0.312
	Non-adenocarcinoma	49 (32.7)	30 (20.0)	19 (12.7)	
**Smoking status**	Smoker	41 (27.3)	26 (17.3)	15 (10.0)	0.222
	Never smoker	109 (72.7)	57 (38.0)	52 (34.7)	
**EGFR status**	Mutation	73 (48.7)	36 (24.0)	37 (24.7)	0.182*
	Wild type	66 (44.0)	40 (26.7)	26 (17.3)	
	Unknown	11 (7.3)	7 (4.7)	4 (2.7)	
**EGFR-TKI**	Erlotinib	97 (64.7)	58 (38.7)	39 (26.0)	0.137
	Gefitinib	53 (35.3)	25 (16.7)	28 (18.7)	
**No. of previous chemotherapy**	1	49 (32.7)	27 (18.0)	22 (14.7)	0.968
	2	101 (67.3)	56 (37.3)	45 (30.0)	
**Tissue specimen**	Resection	37 (24.7)	17 (11.3)	20 (13.3)	0.186
	Biopsy	113 (75.3)	66 (44.0)	47 (31.3)	
**Site of tissue specimen**	Primary tumor	121 (80.7)	68 (45.3)	53 (35.3)	0.663
	Metastatic site	29 (19.3)	15 (10.0)	14 (9.3)	

a ECOG PS: Eastern Cooperative Oncology Group Performance Status Scale

* EGFR unknown patients were not included into the statistical analysis for the limited number

### Efficacy in the whole population

All of the 150 patients were available for response evaluation, including 1 patient with complete response, 51 with partial response, 45 with stable disease, and 53 with progressive disease as their best tumor response. Therefore, the objective response rate (ORR) was 34.7% and the DCR was 64.7%. *EGFR* mutation status and miR-200c expression level were the main factors identified as predicting the disease control to EGFR-TKIs treatment. The ORR and DCR were 57.1% and 84.5% in the patients with *EGFR* unknown and *EGFR* activated mutation subgroup respectively, which is significantly higher than 6.1% (P <0.0001) and 39.4% (P <0.0001) in patients with wild-type *EGFR*. Patients with high level miR-200c expression also showed a significantly higher DCR (74.6% vs. 56.6%, P =0.022) and a numerically higher ORR (38.8% vs. 31.3%, P =0.339) than those with low level.

Survival analyses were performed in all the patients who received EGFR-TKIs therapy with a median follow-up time of 16.7 months (95% CI: 12.6-20.6 months). Among them, 28 patients (18.7%) were still on EGFR-TKIs treatment and 41 (27.3%) were still alive at the last follow-up date of December 30, 2012. The median progression free survival (PFS) and overall survival (OS) for the evaluable patients in this study were 6.6 months (95% CI: 3.6-9.6 months) and 14.1 months (95% CI: 9.7-18.5 months), respectively.

**Figure 7:**
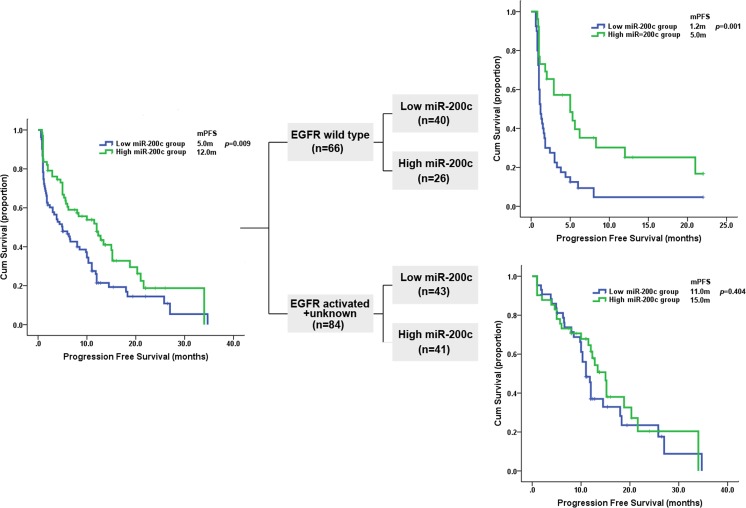
Kaplan-Meier curves showing the probability of progression free survival for patients with NSCLC, according to miR-200c expression level and stratified according to EGFR mutation status.

The PFS in *EGFR-*MUT patients treated with EGFR-TKIs was significantly longer than that of *EGFR-*WT patients (12.0m [95%CI: 10.46-13.54m] vs. 1.7m [95%CI: 1.00-2.40m], P<0.0001). Patients with high level of miR-200c expression (n=67) had longer PFS than those with low-expression (n=83) regardless of *EGFR* status in the whole population (12.0m [95%CI: 7.37-16.63m] vs. 5.00m [95%CI: 1.82-8.18m], P=0.009, Figure 7). Univariate analysis showed lower risk of progression in patients of female, never smoker, ECOG PS≤1, age≥65, *EGFR* activated mutation and high level of miR-200c expression (Table 2). In multivariate analysis, *EGFR* mutations [Hazard ratio(HR): 0.29, 95%CI: 0.19-0.45, P< 0.0001], high level of miR-200c expression (HR: 0.55, 95%CI: 0.36–0.84, P=0.006) and ECOG PS ≤1 (HR: 0.41, 95%CI: 0.21-0.80, P =0.009) remained independent predictors of PFS (Table 2).

**Table 2:** Survival analysis in the whole population

Variables	PFS				OS			
	Univariate		Multivariate		Univariate		Multivariate	
	HR (95%CI)	*p-value*	HR (95%CI)	*p-value*	HR (95%CI)	*p-value*	HR (95%CI)	*p-value*
Gender (Male vs. Female)	1.736 (1.181-2.552)	0.005	1.124 (0.686-1.839)	0.643	1.666 (1.109-2.502)	0.014	1.192 (0.707-2.010)	0.510
Age (≥65 vs. <65)	0.641 (0.415-0.992)	0.046	0.724 (0.457-1.145)	0.167	0.650 (0.403-1.047)	0.077	0.769 (0.461-1.281)	0.313
Smoking status (Yes vs. No)	1.862 (1.242-2.791)	0.003	1.315 (0.800-2.162)	0.279	2.030 (1.328-3.104)	0.001	1.441 (0.848-2.448)	0.176
ECOG PS (0-1 vs. 2-3)	0.260 (0.136-0.497)	<0.0001	0.405 (0.205-0.799)	0.009	0.301 (0.151-0.599)	0.001	0.618 (0.301-1.272)	0.191
EGFR status (mutation vs. wild type)	0.295 (0.196-0.444)	<0.0001	0.291 (0.187-0.452)	<0.0001	0.276 (0.178-0.430)	<0.0001	0.300 (0.189-0.477)	<0.0001
Histology (Non-adeno vs. Adeno)	1.468 (0.987-2.183)	0.058	1.001 (0.643-1.558)	0.998	1.736 (1.128-2.671)	0.012	1.104(0.683-1.784)	0.686
miR-200c expression (High vs. Low level)	0.608 (0.415-0.892)	0.011	0.554 (0.365-0.841)	0.006	0.564 (0.372-0.856)	0.007	0.566 (0.365-0.879)	0.011
Disease stage (IV vs. IIIB)	1.095 (0.599-2.003)	0.768	—	—	1.162 (0.601-2.247)	0.136	—	—
TKIs (Gefitinib vs. Erlotinib)	0.801 (0.534-1.202)	0.284	—	—	1.076 (0.692-1.673)	0.745	—	—
Previous chemotherapy (2 vs.1)	0.888 (0.597-1.321)	0.557	—	—	1.046 (0.684-1.600)	0.834	—	—
Tissues (Biopsy vs. Resection)	1.128 (0.717-1.777)	0.602	—	—	0.955 (0.586-1.556)	0.854	—	—
Biopsy site (Metastatic vs. Primary)	0.763 (0.464-1.254)	0.285	—	—	0.799 (0.467-1.369)	0.415	—	—

As for overall survival, patients with high level of miR-200c expression lived longer than those with low level (21.0m [95%CI: 11.95-30.06m] vs. 10.5m [95%CI: 7.27-13.73m], P =0.006). Female, never smoker, ECOG PS≤1, adenocarcinoma, *EGFR* activated mutation and high level of miR-200c expression were the main predictors for longer OS in univariate analysis. While multivariate analysis found that only *EGFR* activated mutation (HR: 0.30, 95%CI: 0.19-0.48, *p*< 0.0001) and high level of miR-200c expression (HR: 0.57, 95%CI: 0.37-0.88, P =0.011) were still independent predictors for longer overall survival (Table 2).

### High level of miR-200c correlates with longer PFS, longer OS and higher DCR in *EGFR*-WT NSCLC patients

Our data was further analyzed according to the *EGFR* mutation status. When analysis was limited to the *EGFR*-WT subgroup, patients with high miR-200c expression had significantly better clinical outcomes than those with low expression in terms of DCR (57.7% vs. 27.5%, P =0.014), PFS (5.0 m [95%CI: 1.41-8.59] vs. 1.2 m [95% CI: 0.89-1.51), P =0.001, Figure 7], OS (9.6m[95%CI: 4.27-14.93] vs. 5.0m[95%CI: 3.90-6.10], P =0.037) respectively, and a numerically higher ORR (11.5% vs. 2.5%, P=0.292). While no difference in PFS (15.0m[95%CI: 12.46-17.54m] vs. 11.0m[95%CI: 9.62-12.38m], P =0.404; Figure 7), OS (28.0m[95%CI: 20.34-35.66m] vs. 19.0m[95%CI: 15.86-22.14m], P=0.100), DCR(85.3% vs. 83.7%, P =0.835) and ORR(56.1% vs. 58.1%, *p*=0.850) were observed between high and low miR-200c expression cohorts among *EGFR* mutation and unknown group.

In addition, in the subgroup of *EGFR-*WT patients, univariate analysis identified ECOG PS≤1 and miR-200c highly expressed were associated with longer PFS, while multivariate analysis showed that only high level of miR-200c expression could predict a longer PFS(HR: 0.38, 95%CI: 0.21-0.70, P =0.002). For overall survival, both univariate analysis and multivariate analysis demonstrated that high level of miR-200c expression was the only predictor for longer OS (Table 3).

**Table 3:** Survival analysis in the EGFR wild type population

Variables	PFS				OS			
	Univariate		Multivariate		Univariate		Multivariate	
	HR (95%CI)	*p-value*	HR (95%CI)	*p-value*	HR (95%CI)	*p-value*	HR (95%CI)	*p-value*
Gender (Male vs. Female)	1.833 (0.981-3.425)	0.057	1.431 (0.691-2.963)	0.335	1.776 (0.930-3.394)	0.082	1.771 (0.917-3.421)	0.089
Smoking status (Yes vs. No)	1.577(0.927-2.685)	0.093	1.466 (0.761-2.824)	0.252	1.422 (0.823-2.458)	0.207	—	—
ECOG PS (0-1 vs. 2-3)	0.433 (0.207-0.905)	0.026	0.503 (0.230-1.098)	0.085	0.635 (0.293-1.375)	0.249	—	—
miR-200c expression (High vs. Low level)	0.408 (0.229-0.725)	0.002	0.384 (0.211-0.698)	0.002	0.555 (0.315-0.979)	0.042	0.538 (0.303-0.957)	0.035
Biopsy site (Metastatic vs. Primary)	1.465 (0.689-3.114)	0.321	—	—	1.970 (0.956-4.059)	0.066	1.685 (0.808-3.513)	0.164
Age (≥65 vs. <65)	0.600 (0.301-1.195)	0.146	—	—	0.673 (0.344-1.319)	0.249	—	—
Histology (Non-adeno vs. Adeno)	0.918 (0.542-1.555)	0.750	—	—	0.838 (0.488-1.440)	0.522	—	—
Disease stage (IV vs. IIIB)	1.532 (0.610-3.852)	0364	—	—	1.494 (0.593-3.759)	0.394	—	—
TKIs (Gefitinib vs. Erlotinib)	0.713 (0.402-1.264)	0.247	—	—	0.674 (0.370-1.226)	0.196	—	—
Previous chemotherapy (2 vs.1)	0.854 (0.484-1.505)	0.585	—	—	0.936 (0.525-1.670)	0.824	—	—
Tissues (Biopsy vs. Resection)	1.441 (0.774-2.683)	0.250	—	—	0.946 (0.505-1.772)	0.861	—	—

## DISCUSSION

In this study, we observed that miR-200c regulated EMT by targeting ZEB1 in NSCLC cell lines and high expression of miR-200c can increase sensitivity to gefitinib. Additionally, resistance to gefitinib induced by miR-200c down regulation implicated the activation of PI3K/AKT and MEK/ERK pathways. Finally, our clinical results showed that in *EGFR-*WT patients with high miR-200c expression could benefit more from EGFR-TKIs than those with low miR-200c expression, while no such effect was observed in the *EGFR-*MUT subgroup. This is the first comprehensive study to have found that miR-200c overexpression might be a potential predictive biomarker for the outcome of EGFR-TKIs in advanced NSCLC patients with *EGFR-*WT.

Several phase III clinical trials have showed that *EGFR* mutation was a main predictor for the efficacy of EGFR-TKIs [4, 23]. However, there is about 15% of Caucasian and 30%-40% of Asian NSCLC patients harbor *EGFR-*MUT, and the majority of these patients are *EGFR-*WT [13, 24]. As for patients with *EGFR-*WT, several studies had been performed to confirm whether EGFR-TKIs worked in this subgroup of NSCLC patients. The TAILOR trial [11] from Italy, DELTA [25] from Japan and CTONG0806 [12] from China indicated chemotherapy is superior to EGFR-TKIs as a second-line therapy in patients with *EGFR-*WT. However, on the other side, we also found that a part of these patients could also benefit from targeted therapy. The ORR and PFS of EGFR-TKIs were 3-14.7% and 1.3-2.4 months, while the DCR reached as high as 52.8% in the DELTA trial, which indicates that a considerable part of *EGFR*-WT patients could benefit from EGFR-TKIs.

The EGFR protein expression and gene copy number are the most commonly evaluated biomarkers in the *EGFR*-WT subgroup [26]. A phase II trial from China showed that erlotinib is not superior to pemetrexed as a second-line therapy in *EGFR-*WT NSCLC patients who are EGFR FISH-positive [27]. Further, the meta-analysis data from SATURN and BR.21 showed that EGFR IHC-positive or FISH-positive was not strong enough to select suitable patients for EGFR-TKIs therapy[28]. Recently, the PROSE study, which used VeriStrat test to predict the efficacy of EGFR-TKIs, demonstrated *EGFR*-unknown or *EGFR-*WT NSCLC patients with VeriStrat “good” classification would have similar efficacy in second-line therapy using either chemo or EGFR-TKIs[29]. However, larger prospective studies are needed to confirm these results. There are no validated and reproducible biomarkers to identify proper *EGFR-*WT patients to receive EGFR-TKIs treatment.

MiRNAs play key roles in carcinogenesis and the maintenance of malignant phenotypes. Their aberrant expression can affect clinical efficacy of chemotherapy or even targeted therapy in several cancers. Our previous research showed that overexpression of miR-21 and miR-214 are associated with acquired resistance of EGFR-TKIs in NSCLC [30, 31]. In this study, our results demonstrated that elevated miR-200c could reverse the phenotype of NSCLC cell lines from mesenchymal to epithelial. We validated that ZEB1 was the target of miR-200c in NSCLC cell lines, which was in line with the other studies [21, 22]. Previous studies have shown that persistent activations of PI3K/AKT and MEK/ERK pathways are considered as two crucial mechanisms leading to EGFR-TKIs resistance [32, 33]. Meanwhile, PI3K/AKT pathway also plays a vital role in EMT and PI3K inhibitors and can reverse the progression of EMT [34, 35]. This study showed that upregulated miR-200c could partially resensitize the primary resistant NSCLC cell lines to gefitinib and suppress the activation of PI3K/AKT and MEK/ERK pathways. All the results mentioned above showed that close relationships exist between miR-200c, EMT, PI3K/AKT and MEK/ERK signal pathways, which contribute to the resistance of EGFR-TKIs in NSCLC.

This is the first study to demonstrate that miR-200c overexpression might be an independent predictor of longer PFS and OS together with superior DCR in NSCLC patients with *EGFR-*WT. We found that patients with a higher level of miR-200c expression had a significantly higher DCR (57.7% vs. 27.5%, P=0.014), longer PFS (5.0 vs. 1.2 months, P =0.001), longer OS (9.6 vs. 5.0 months, P =0.037) and numerical higher ORR (11.5% vs. 2.5%, P =0.292) compared with those with lower expression. The same significant difference could not be shown in the *EGFR-*MUT subgroup, suggesting that miR-200c may be only a predictive marker for EGFR-TKIs efficacy in *EGFR-*WT patients. We also found that patients with *EGFR* mutations were more likely to have a miR-200c overexpression.

With the development of molecular subtype in lung cancer, NSCLC patients with *EGFR-*WT had been identified to carry several new “driver mutations”, including *KRAS*, *HER2*, and *BRAF* mutations, as well as *ALK*, *ROS1*, and *RET* gene fusion. Recent evidences presented *BRAF* and *KRAS* oncogenes are involved in the TGF-β1 pathway which play an important role in induction and maintenance of EMT in colon carcinoma [36]. Another study on circulating tumor cells (CTCs) in lung cancer suggested that homogeneous mesenchymal phenotype was observed in *ALK*-rearranged CTCs, which indicated EMT may be triggered by *ALK* tyrosine kinase activation and then promotion of tumor cell migratory properties [37]. EMT appears to be a potential factor behind the “driver mutation” that influences the sensitivity to EGFR-TKIs. Recently, our study found that, comparing with mesenchymal phenotype, epithelial phenotype was associated with a significantly higher ORR, longer PFS and OS after EGFR-TKIs therapy in advanced NSCLC patients with *EGFR*-WT [38]. Similar results were also observed in the biomarker analysis in BATTLE study. While, clarifing the epithelial or mesenchymal phenotype should have enough tumor tissue and using the complex laborious procedures of immunohistochemistry with complicated evaluation standards and subjective assessment of pathologists. MiR-200c as the main contributor to EMT could reverse resistance to gefitinib in this study, which may become a suitable and promising surrogate to judge the phenotype of tumor.

In conclusion, we found that miR-200c over-expression could reverse the mesenchymal phenotype of cells to epithelial phenotype by targeting ZEB1 in NSCLC, which could also re-sensitize EGFR-TKIs in NSCLC cell lines with primary resistance. Moreover, miR-200c over-expression could predict a better efficacy of EGFR-TKIs in advanced NSCLC patients with *EGFR*-WT. Our finding warrant further clinical studies to investigate the role of miR-200c expression in guiding the tailored EGFR-TKIs therapy in advanced NSCLC patients with *EGFR*-WT.

## MATERIALS AND METHODS

### Cell line and cell culture

Seven human NSCLC cell lines, including PC9, PC9/R, H23, A549, H1975, H460 and H1299, were cultured in Dulbecco's modified Eagle medium (DMEM) (Hyclon, Longan, UT) containing 10% fetal bovine serum (FBS) (Life Technologies, Grand Island, NY) with 100 units/mL penicillin and 100 μg/mL streptomycin at 37°C with 5% CO_2_. The characteristics and IC50 of these cell lines to gefitinib are listed in Supplementary Table S1.

### RNA isolation and quantitative reverse-transcriptase polymerase chain reaction for miR-200c

Total RNA was extracted from cells using TRIzol Reagent (TaKaRa, Shiga, Japan), and total miRNA of FFPE tissues was extracted by miRNeasy FFPE kit (QIAGEN, Hilden, Germany). miR-200c and U6-specific cDNA were synthesized using gene-specific primer. Reverse transcription was performed using the RevertAid First Strand cDNA Synthesis Kit(Thermo scientific, Rockford, IL) according to the manufacturer's instructions. miR-200c was quantified in Stratagene Mx3000P™ (Agilent Technologies, Palo Alto, CA) by reverse-transcriptase polymerase chain reaction (qRT-PCR) using SYBR Premix Ex Taq (TaKaRa) and normalized to U6 according to the manufacturer's protocols. All qRT-PCRs were performed in duplicate. Primers were listed in Supplementary Table S2. Relative quantification of miR-200c expression was calculated using the 2^−ΔCT^ formula.

The cut-off point for miR-200c expression in our study was determined from the “minimum P value” and lowest Hazard ratio for PFS and OS from every 5 percentile of 2^−ΔCT^ of miR-200c expression. The best cut-off value to predict both PFS and OS (Supplementary Table S4) was fixed at the 55th percentile (2^−ΔCT^=0.01385). MiR-200c was defined as high expression when 2^−ΔCT^≥0.01385 and as low expression when 2^−ΔCT^<0.01385.

### Cell growth inhibition assay

The cells were seeded onto 96-well plates at a density of 5×10^3^ overnight. Gefitinib (AstraZeneca, Luton, UK) was added in a dose-dependent manner and the cells were incubated for 72h. After adding 20μL of MTT (5mg/mL) into each well, cells were incubated at 37°C for 4h. Finally, the supernatant was discarded and 200μL of DMSO was added to each well to dissolve the precipitate. The optical density was assessed at 570 nm using a 96-well microplate reader (Bio-rad, Hercules, CA). Each experiment was repeated in triple. IC_50_ was defined as the concentration needed for 50% reduction in the absorbance.

### Transfection of miR-200c inhibitor

MiR-200c inhibitor and its negative control oligonucleotides (Supplementary Table S3) were obtained from Invitrogen (Carlsbad, CA). Cells were planted into 6-well plates (2×10^5^ cells/well) and transfected with miRNA inhibitor using Lipofectamine™ 2000 (Invitrogen) according to the manufacturer's protocol. After 72 h, qRT-PCR, MTT assay, and western blot analysis were performed.

### Lentivirus Infection

Lentivirus (8×10^8^ TU/mL) packaging of green fluorescent protein (GFP) LV-hsa-mir-200c and negative control (NC) were constructed in Genechem (Shanghai, China). Cells (1×10^3^) were plated on to 96-well plates 24h before LV-hsa-mir-200c and negative control were added to infected H1975, A549 and H1299 at different volumes to determine the best multiplicity of infection (MOI) value at which concentrations no virus toxicity effect on cells was found. 96h after infection, the GFP gene expression was observed under fluorescence microscope, and the infected cells were collected for subsequent culture. The experiment was repeated three times.

### Western blot analysis

Proteins were extracted using ReadyPrep Total Protein Extraction Kit (Bio-rad). Protein extracts (25μg) were separated by 10% SDS-PAGE and then transferred onto nitrocellulose membranes (Pall, Port Washington, NY) using a Bio-Rad Tetra blotting module. The membranes were blocked in TBS that contained 5% nonfat milk powder for 2h before incubation with rabbit monoclonal antibody diluted in TBS/5% nonfat milk powder overnight at 4°C. Subsequently membranes were washed with TBST and incubated for 2h with horseradish peroxidase (HRP) conjugated goat anti-rabbit IgG (1:2500, KPL, Gaithersburg, MD) at room temperature. All the rabbit monoclonal antibodies were purchased from Epitomics (Burlingame, CA), including Akt1 phospho (pS473) (1:2000), Akt1 (1:10000), Vimentin (1:5000), E-cadherin (1:5000) and ZEB1 (1:2000). Rabbit anti-GAPDH antibody (1:2500) was detected simultaneously as a loading control. Specific proteins were detected using an ECL kit (Thermo scientific) and Gel doc XR (Bio-rad). Protein band densities were calculated by using Quantity one (Bio-rad). Densitometry results were normalized against GAPDH expression.

### Immunocytochemistry

In the previous day, 10^5^ cells were plated on 24 wells containing glass coverslips. The coverslips were washed three times by PBS, and then cells were fixed by paraformaldehyde for 30 min and treated with Triton X-100 to increase permeability of cell membrane. Then cells were washed thrice in PBS, followed by blocking of the endogenous peroxidase by 3% hydrogen peroxidase for 15 min and 5% nonfat milk powder for 2h to block nonspecific binding. Subsequently cells were incubated with diluted primary antibodies (1:150) at 4°C overnight. After washing 3 times in PBS, cells were incubated with HRP-conjugated secondary antibody (1:100; KPL) for 1 h at room temperature. Immunoreactivity was demonstrated using diaminobenzadine (Invitrogen) for increased sensitivity. Sections were counterstained with hematoxylin and covered with a cover glass. The negative controls were incubated with a solution that was devoid of any primary antibody.

### Patients and sample collection

NSCLC patients in advanced stage who received EGFR-TKIs (gefitinib or erlotinib) as second- or third-line therapy from September 2008 to December 2012 were included in the study. All patients had formalin-fixed paraffin-embedded (FFPE) samples for *EGFR* mutation status and miRNA expression analysis. The tumor tissues were fixed in 10% neutral-buffered formalin, and stored as paraffin-embedded archival until use. All tissues were reviewed by experienced pathologists for confirmation of histological type and tumor content of >30%. Paraffin sections were then cut at a thickness of 5 μm. DNA and RNA were extracted from 6 pieces of the paraffin-embedded slides. Objective tumor response was determined using Response Evaluation Criteria in Solid Tumors (RECIST version 1.1). The patients underwent computed tomography scan covering target lesions 4-6 weeks after the initiation of EGFR-TKIs treatment and then every 8 weeks or when indicated by symptoms. This study was approved by the Ethics Committee of Shanghai Pulmonary Hospital, Tongji University, and written informed consent was obtained from each participant before the initiation of any study-related procedures.

### EGFR mutation analysis

*EGFR* mutation status was detected in Tongji University Medical School Cancer Institute (Shanghai, China). Briefly, total DNA was extracted using QIAmp DNA FFPE tissue kit (QIAGEN) according to the manufacturer's protocol. *EGFR* mutation detection was performed using a Human EGFR Gene Mutations Fluorescence PCR Diagnostic Kit (Amoy Diagnostics Company Ltd., Xiamen, China), which is based on ARMS technology. The assay can identify 29 most common types of *EGFR* mutations (exon18-21) currently described in lung cancer. All experiments were conducted according to the user manual from the manufacturer. PCR reaction was performed using Stratagene Mx3000P™ (Agilent Technologies). The details were described in our previous articles [39, 40].

### Statistical analysis

We used the SPSS statistical software package (version 17.0; SPSS, Inc., Chicago, IL) to perform the statistical analysis. Differences of miR-200c expression between two groups were assessed by the Mann-Whitney U test or Student's t test. A Chi-square test or Fisher exact test was used to analyze the association between miR-200c expression level and clinical characteristic variables, ORR and DCR. PFS was defined as the period from the date of EGFR-TKIs administration to the date of disease progression or death. OS was calculated as the time from the beginning of therapy to death or the last follow-up date. All time-to-event outcomes were estimated using the Kaplan-Meier method and compared across groups with the log-rank test or the Cox proportional hazards model. All statistical tests were two-sided, and statistical significance was defined as P<0.05.

### SUPPLEMENTARY TABLES


