# Supplementary material for: MiR-200c overexpression is associated with better efficacy of EGFR-TKIs in non-small cell lung cancer patients with EGFR wild-type
Source: Oncotarget. 2014 Aug 8;5(17):7902–16. doi: 10.18632/oncotarget.2302 (PMC4202169; doi:10.18632/oncotarget.2302)
Supplement: Supplementary file 1 [file oncotarget-05-7902-s001.pdf]

SUPPLEMENTARY TABLES

Supplementary Table S1. Cell characteristics

Cell	Histology	EGFR status	Kras status	Other	IC_{50}(μM) to gefitinib
PC9	Adenocacinoma	del exon 19	WT	/	0.028
PC9/R	Adenocacinoma	del exon 19	WT	/	10.778
A549	Adenocacinoma	WT	Mut exon1	/	23.571
H1299	Adenocarcinoma	WT	WT	p53 null	35.882
H23	Adenocarcinoma	WT	Mut exon1	/	24.953
H460	Large-cell carcinoma	WT	Mut exon2	/	13.711
H1975	Adenocarcinoma	L858R/T790M	WT	/	15.062

Supplementary Table S2. Sequence of primer

Primer	Sequence
U6-RT	5′-GTCGTATCCAGTGCAGGGTCCGAGGTATTCGCAC TGGATACGACAAAATATGGAAC-3′
U6-forward	5′-TGCGGGTGCTCGCTTCGGCAGC-3′
U6-reverse	5′-CCAGTGCAGGGTCCGAGGT-3′
miR-200c-RT	5′-GTCGTATCCAGTGCAGGGTCCGAGGTATTCGCAC TGGATACGACTCCATC-3′
miR-200c-forward	5′-TAATACTGCCGGGTAAT-3′
miR-200c-reverse	5′-GTGCAGGGTCCGAGGT-3′
ZEB1-forward	5′-TTCAAACCCATAGTGGTTGCT-3′
ZEB1-reverse	5′-TGGGAGATACCAAACCAACTG-3′
GAPDH-forward	5′-GAAATCCCATCACCATCTTCCAGG-3′
GAPDH-reverse	5′-GAGCCCCAGCCTTCTCCATG-3′

Supplementary Table S3. Sequence of miRNA inhibitor

	Sequence
hsa-miR-200c-3p inhibitor	5′-UCCAUCAUUACCCGGCAGUAUUA -3′
MicroRNA inhibitor N.C	5′-CAGUACUUUUGUGUAGUACAA-3′

Supplementary Table S4. Cut-off point analysis of miR-200c expression

Percentile	Cut-off point ($2^{-\Delta CT}$ of miR-200c expression)	PFS			OS		
		P-value	HR(95% CI)	*Log-rank* P-value	P-value	HR(95% CI)	*Log-rank* P-value
5%	0.00043						
10%	0.00067						
15%	0.01172	0.312	1.332 (0.764–2.324)	0.303	0.290	1.392 (0.754–2.570)	0.284
20%	0.00160	0.774	0.934 (0.585–1.490)	0.771	0.572	1.208 (0.721–2.025)	0.468
25%	0.00258	0.273	0.786 (0.511–1.209)	0.264	0.906	0.972 (0.611–1.574)	0.905
30%	0.00386	0.170	0.755 (0.505–1.128)	0.162	0.597	0.891 (0.580–1.368)	0.594
35%	0.00531	0.187	0.770 (0.522–1.135)	0.179	0.411	0.841 (0.556–1.271)	0.406
40%	0.00721	0.091	0.721 (0.494–1.052)	0.084	0.251	0.789 (0.527–1.182)	0.246
45%	0.00917	0.057	0.695 (0.478–1.011)	0.052	0.066	0.687 (0.460–1.026)	0.062
50%	0.01122	0.015	0.628 (0.431–0.914)	0.013	0.011	0.592 (0.394–0.888)	0.010
**55%**	**0.01385**	**0.011**	**0.608 (0.415–0.892)**	**0.009**	**0.007**	**0.564 (0.372–0.856)**	**0.006**
60%	0.01694	0.054	0.682 (0.462–1.006)	0.048	0.037	0.635 (0.415–0.972)	0.033
65%	0.02266	0.021	0.619 (0.412–0.932)	0.018	0.013	0.563 (0.359–0.885)	0.011
70%	0.02852	0.100	0.697 (0.453–1.072)	0.093	0.089	0.665 (0.415–1.064)	0.084
75%	0.04595	0.052	0.628 (0.393–1.004)	0.046	0.054	0.603 (0.361–1.008)	0.049
80%	0.06173	0.044	0.587 (0.350–0.987)	0.039	0.089	0.610 (0.345–1.078)	0.083
85%	0.09446	0.026	0.476 (0.248–0.914)	0.020	0.082	0.542 (0.272–1.080)	0.074
90%	0.20377						
95%	0.34736						
